# Association between Interleukin 35 Gene Single Nucleotide Polymorphisms and Systemic Lupus Erythematosus in a Chinese Han Population

**DOI:** 10.3390/biom9040157

**Published:** 2019-04-22

**Authors:** Shi-Yang Guan, Li-Na Liu, Yan-Mei Mao, Chan-Na Zhao, Qian Wu, Yi-Lin Dan, Napoleon Bellua Sam, Hai-Feng Pan

**Affiliations:** 1 Department of Epidemiology and Biostatistics, School of Public Health, Anhui Medical University, 81 Meishan Road, Hefei 230032, China; gsy92@foxmail.com (S.-Y.G.); pachyrhizus123@sina.com (L.-N.L.); mym199507@163.com (Y.-M.M.); zcn19940602@163.com (C.-N.Z.); qianwu1994@163.com (Q.W.); danyilin98@163.com (Y.-L.D.); belluadsm@yahoo.com (N.B.S.); 2 Anhui Province Key Laboratory of Major Autoimmune Diseases, Hefei 230032, China

**Keywords:** interleukin-35, systemic lupus erythematosus, gene single nucleotide polymorphisms, autoimmune diseases

## Abstract

Interleukin-35 (IL-35) exerts crucial roles in the pathogenesis and development of systemic lupus erythematosus (SLE), in this study we aim to explore the associations between IL-35 gene polymorphisms and the susceptibility, clinical features and plasma IL-35 levels of SLE patients, respectively. 490 SLE patients and 489 healthy controls were recruited in our study. The correlations between the polymorphisms of seven SNPs of IL-35 encoding gene and the susceptibility, main clinical manifestations of SLE were evaluated, respectively. Plasma IL-35 levels were assessed in 76 SLE patients, and the associations between plasma IL-35 levels and the polymorphisms of genotyped SNPs were explored. There were significant associations between the polymorphisms of rs4740 and the occurrence of renal disorder, hematological disorder in SLE patients, respectively (*p* = 0.001; *p* = 0.001). In addition, there were no significant associations observed between the genotype frequencies of genotyped SNPs and the risk of SLE, plasma IL-35 levels, respectively. The polymorphism of rs4740 of IL-35 encoding gene is associated with the occurrence of renal disorder and hematological disorder of SLE patients.

## 1. Introduction

Systemic lupus erythematosus (SLE) is a prototypic autoimmune disease that is characterized by the impaired immune tolerance of the immune system and the massive production of autoantibody, and eventually leads to a variety of tissues’ and organs’ functions being damaged, such as skin, blood vessels, kidney, etc [1,2,3,4]. It is more prevalent in women than men, and its diverse manifestations and disease heterogeneity pose great challenges to the physical and mental health of women of childbearing age. [2,5,6]. Although significant advances have been made in the current studies of SLE, the etiology and pathogenesis of the disease remain unclear, and generally believe to be the result of the interaction of environmental, genetic and immune factors [7,8,9,10,11].

Currently, the imbalance between Treg cells and Th17 cells is considered to be an essential immunopathogenesis of multiple autoimmune diseases [12,13,14,15,16,17,18]. Interleukin 35 (IL-35), the latest identified cytokine of interleukins 12 (IL-12) family, is consisted of two subunits (p35 and EBI3) [19,20,21] and secreted by Treg cells [22,23] and activated B cells [21,24], etc. Studies revealed that IL-35 facilitates the differentiation and optimal immunosuppression of Treg cells [22,23], and restrains the propagation and function of Th17 cells [25,26,27]; the levels of IL-35 and IL-35 mRNA are significantly elevated in SLE patients [28], and IL-35 can significantly relieve lupus flare and lupus nephritis, and reduce the plasma levels of proinflammatory cytokines (IFN-γ, TNF-α, IL-6 and IL-17A) and elevate the plasma levels of anti-inflammatory cytokines (IL-10 and IL-2) in MRL(Murphy Roths Large)/LPR mice [28]. These findings suggest that IL-35 exerts crucial roles in the pathogenesis and development of SLE.

The encoding gene of p35 subunit, *IL-12A*, is located on chromosome 3q25.33, and several *IL-12A* polymorphisms were associated with the susceptibilities to primary biliary cirrhosis (PBC), Graves’ disease and Alzheimer’s disease [29,30,31]; *EBI3*, the encoding gene of another subunit, EBI3, is located on chromosome 19q13.3, and the polymorphisms of *EBI3* rs428253, rs4740 and rs4905 were associated with the decreased risk of developing ulcerative colitis (UC) [32]. Considering there is no evidence about the effects of IL-35 gene polymorphisms on SLE, in this case-control study we explored the associations between IL-35 gene polymorphisms and the susceptibility, main clinical manifestations, plasma IL-35 levels of SLE patients in a Chinese Han population, respectively. 

## 2. Materials and Methods 

### 2.1. Subjects

All the participants included in our study were of Chinese Han ancestry. A total of 490 SLE patients were recruited in our study from Department of Rheumatism and Immunity, the First Affiliated Hospital of Anhui Medical University and Department of Rheumatology and Immunology, Anhui Provincial Hospital, all the SLE patients met the criteria for the classification of SLE (American College of Rheumatology, 1997), and renal disorder of SLE was defined if any one of following definitions was satisfied: (a) Persistent proteinuria > 0.5 g/d or > 3+ if quantitation not performed; (b) Cellular casts: may be red blood cell, hemoglobin, granular, tubular, or mixed [33]. 489 healthy controls were selected from the Health Examination Center, the First Affiliated Hospital of Anhui Medical University, all the healthy controls eligible to the inclusion criteria (a. do not meet any of the criteria for SLE classification, b. self and immediate relatives have no previous history of autoimmune diseases, c. match with SLE patients by age and gender). The informed consent was obtained from all the participants, and demographic profiles and clinical characteristics were collected from the participants. This study was approved by the Medical Ethics Committee of Anhui Medical University.

### 2.2. SNP Selection, Genotyping and Enzyme-Linked Immunosorbent Assay (ELISA)

The single nucleotide polymorphisms (SNPs) of IL-35 encoding gene, *IL-12A* and *EBI3*, were selected from HapMap database of Han Chinese population (CHB) in Beijing (HapMap Data Rel 24/phaseII Nov08, on NCBI B36 assembly, dbSNP b126) with minor allele frequency (MAF) ≥ 0.05. The pair-wise linkage disequilibrium (LD) between SNPs with r^2^ threshold of 0.8 was calculated by Haploview software (http://www.broad.mit.edu/haploview/haploview) [34]. Eventually, seven tag SNPs were included in our study, including rs2227314, rs2243115, rs2243123, rs2243131 in *IL-12A* gene, and s428253, rs4740, rs9807813 in *EBI3* gene, the observed genotype frequencies of all genotyped SNPs were consistent with that of expected ones in healthy controls (all *p* > 0.05) (Table 1), and further genotyped by high-throughput SNPscan^TM^ genotyping assays (Genesky Biotechnologies Inc., Shanghai, China). Plasma IL-35 levels were assessed by Human IL-35 ELISA kits according to the manufacturer’s instruction (R & D Systems, Inc. Minneapolis, MN, USA).

### 2.3. Statistical Analysis

Statistical analysis was performed by the Statistical Package for the Social Science (SPSS) version 23.0 for Windows. Shapiro-Wilk test was implemented for testing the normality of data, continuous data was described as mean ± SD for normally distributed data or median (interquartile range, IQR) for non-normally distributed data, and categorical data was presented as frequencies and percentages. The difference of continuous data was evaluated by *t*-test, F-test or Mann-Whitney U test, and that of categorical data was fulfilled by chi-square test or Fisher exact test. Hardy-Weinberg equilibrium (HWE) was tested to compare the frequencies of observed genotype to that of expected ones [35], and haplotype analyses were completed using online platform SHEsis (http://analysis.bio-x.cn/myAnalysis.php) [36]. A two-sided *p* value less than 0.05 was considered as statistically significant.

## 3. Results

### 3.1. Demographic Characteristics, Clinical Features of Subjects

The demographic characteristics of all participants are presented in Table 2, there were no significant differences in age and gender between SLE patients and healthy controls, respectively (*t* = 1.324, *p* = 0.186; χ^2^ = 0.096, *p* = 0.757). The clinical characteristics of SLE patients are illustrated in Table 2, the major clinical features of SLE patients were butterfly rash (45.5%), arthritis (49.2%), hematological disorder (68.0%) and immunological disorder (73.3%).

### 3.2. Association of IL-35 Gene Polymorphisms with Risk of SLE

The allele frequencies and genotype frequencies of seven genotyped SNPs in SLE patients and healthy controls were presented in Table 3, there were no significant differences in allele frequencies and genotype frequencies between SLE patients and healthy controls in all seven genotyped SNPs (rs2227314, rs2243115, rs2243123, rs2243131, rs428253, rs4740 and 9807813, all *p* > 0.05). Furthermore, we explored the IL-35 gene polymorphisms with risk of SLE under dominant, recessive and additive model, and there were also no significant differences in IL-35 gene polymorphisms between SLE patients and healthy controls in all seven genotyped SNPs (all *p* > 0.05) (Table 3).

### 3.3. Association of IL-35 Gene Polymorphisms with the Clinical Features of SLE Patients

The associations between allele frequencies and genotype frequencies of seven genotyped SNPs and main clinical features of SLE patients were detailed in Supplementary Tables (Tables S1 to S7). There were significant differences in both genotype frequency and allele frequency of rs4740 between SLE patients with renal disorder and those without (χ^2^ =13.759, *p* = 0.001; χ^2^ = 11.804, *p* = 0.001); and there was a significant difference in genotype frequency of rs4740 between SLE patients with hematological disorder and those without (χ^2^ = 6.683, *p* = 0.036). However, there were no significant associations between genotype frequencies and allele frequencies of the other six SNPs (rs2227314, rs2243115, rs2243123, rs2243131, rs428253 and rs9807813) and the clinical features of SLE patients.

### 3.4. Association of Plasma IL-35 Levels with IL-35 Genotypes in SLE Patients 

We randomly selected 76 plasma samples from 490 SLE patients and assessed their plasma IL-35 levels, the results revealed that there are no significant differences of plasma IL-35 levels in different IL-35 genotypes of each SNP, respectively (Table 4).

### 3.5. Haplotype Analyses

Five main haplotypes (GGTA, GTTA, TTCA, TTTA and TTC) were constructed for rs2227314, rs2243115, rs2243123 and rs2243131 in *IL-12A* locus, and five main haplotypes (CAC, CGC, GAC, GAT and GGC) were constructed for rs428253, rs4740 and rs9807813 in *EBI3* locus. There were no significant differences in the frequency of each constructed haplotype in SLE patients and healthy controls (Table 5 and Table 6).

## 4. Discussion

The disturbances in balance between Treg cells and Th17 cells have been considered as a new paradigm for pathogenesis of autoimmune diseases [3,18,37]. IL-35 as a newly identified cytokine of IL-12 family exerts an indispensable role in the balance of Treg cells and Th17 cells [4,38]. IL-35 abnormally elevated in SLE patients [39], and significantly alleviated lupus flare, lupus nephritis and plasma levels of proinflammatory cytokines in MRL/lpr mice [28]; there was an inverse correlation between serum IL-35 levels and disease activity in rheumatoid arthritis (RA) patients [40], and IL-35 significantly relieved the synovial hypertrophy and bone destruction of RA model [41]; serum IL-35 were significantly over-expressed in systemic sclerosis (SSc) patients [42], and the frequency of Treg cells, the major source of IL-35, was associated with clinical phenotype and progression of SSc patients [43]; IL-35 levels significantly reduced in inflammatory bowel diseases (IBD) patients and idiopathic thrombocytopenic purpura (ITP) patients [44,45], exhibited a negative association with disease activity of IBD patients and a positive association with platelet counts in active ITP patients [44,45]. All of this evidence implies that IL-35 is involved in the pathogenesis and progression of autoimmune diseases.

Several studies have explored the relations between the gene single nucleotide polymorphisms of interleukin 35 encoding genes (*IL-12A* and *EBI3*) and susceptibility to autoimmune diseases: genome-wide association analysis revealed that genetic variants of rs6441286, rs574808 in *IL-12A* locus were significantly associated with the PBC in North American white subjects [29]; Guo et al. [30] reported that the high frequency of allele A of *IL-12A* rs568408 related to the high Graves’ disease risk in two Chinese cohort ; *IL-12A* rs568408 was significantly associated with the risk of late-onset Alzheimer’s disease (LOAD), *IL-12A* rs2243115 elevated LOAD risk only in Apolipoprotein E, type ϵ4 allele carriers and genotype frequencies of *IL-12A* rs568408 had no significant associations with the risk of RA in Han Chinese populations [31,46]; there were significant relations between *EBI3* rs428253 and protective effects against allergic rhinitis in Chinese subjects [47]; rs428253, rs4740 and rs4905 in *EBI3* locus significantly correlated with the decreased UC risk in the Mexican population [32]. 

In this study, we explored the associations between IL-35 gene single nucleotide polymorphisms with genetic susceptibility to SLE in a Chinese Han population. There were significant associations observed between the polymorphisms of rs4740 and the occurrence of renal disorder and hematological disorder in SLE patients, respectively, which echoed previous studies that rs4740 polymorphism exhibits protective effects on UC and pulmonary tuberculosis in Mexican and Chinese populations, respectively [32,48]. Lupus nephritis, as one of the most devastating complications of SLE, predicts poor long-term outcomes with more than four-fold increase in mortality [49]. In US, at the time of diagnosis around 35% SLE patients have clinical evidence of nephritis, within a decade of the illness estimated total of 50–60% patients develop nephritis [50]. In the present study, the prevalence of renal disorder in SLE patients was 37.6% with median disease duration of 4.06 years. Previous study has suggested that serum IL-35 could be served as a potential biomarker of renal involvement in SLE patients; the serum levels of IL-35 were significantly lower in nephritis patients with higher levels of serum creatinine, blood urea nitrogen and blood uric acid [51]. Our result adds novel evidence in elucidating the genetic underpinnings driving nephritis among SLE patients. However, there were no significant differences in both genotype frequencies and allele frequencies of all seven genotyped SNPs (rs2227314, rs2243115, rs2243123, rs2243131, rs428253, rs4740 and rs9807813) between SLE patients and healthy controls, and no significant associations between the polymorphisms of other genotyped SNPs (rs2227314, rs2243115, rs2243123, rs2243131, rs428253 and rs9807813) and the occurrence of main clinical manifestations in SLE patients. Posadas-Sánchez et al. [52] illustrated that in healthy controls, IL-35 serum levels are significant different among different genotypes of rs4740 and rs4905, respectively. In this study, ELISA results demonstrated that plasma IL-35 levels have no significant association with genotype polymorphisms of all genotyped SNPs. 

As far as we know, this is the first study explored about the associations between IL-35 gene single nucleotide polymorphisms and the genetic susceptibility to autoimmune diseases, in which IL-35 encoding genes, *IL-12A* and *EBI3*, were analyzed together. However, one limitation of our study should be considered, i.e., there was no information about the medication taking history of SLE patients and some of the SLE patients may have been treated with steroids or immunosuppressive agents, which potentially concealed the actual associations between plasma IL-35 levels and IL-35 gene single nucleotide polymorphisms, so further studies with known medication history of participants are needed to verify the results. 

## 5. Conclusions

In conclusion, there is a significant association between the polymorphism of rs4740 and the occurrence of certain clinical manifestations in SLE patients, providing new clues for the prevention, diagnosis and treatment of SLE.

## Figures and Tables

**Table 1:** SNPs genotyped in *IL-12A* and *EBI3* genes.

Gene	SNP ID	Chr	Chr Position	Allele	mRNA	Region	*p* Value for HWE Test
*IL-12A*	rs2227314	3	159712054	G/T	NM_000882.3	Intron 6	0.902
*IL-12A*	rs2243115	3	159706280	G/T	NM_000882.3	5′ Flanking	0.908
*IL-12A*	rs2243123	3	159709651	C/T	NM_000882.3	Intron 2	0.891
*IL-12A*	rs2243131	3	159712058	A/C	NM_000882.3	Intron 6	0.954
*EBI3*	rs428253	19	4229913	C/G	NM_005755.2	Intron 1	0.262
*EBI3*	rs4740	19	4236996	A/G	NM_005755.2	Exon 5	0.887
*EBI3*	rs9807813	19	4232415	C/T	NM_005755.2	Intron 2	0.322

Chr, Chromosome; HWE, Hardy-Weinberg equilibrium.

**Table 2:** Demographic characteristics and clinical features of participants.

Variable	SLE Patients (n = 490)	Healthy Controls (n = 489)	*t/χ^2^*	*p* Value
Age (years)	37.57 ± 11.49	38.53 ± 11.31	1.324 *	0.186
Gender, n (%)				
Male	56 (11.4)	59 (12.1)	0.096 **	0.757
Female	434 (88.6)	430 (88.0)		
Disease duration, years	4.06 (1.05–8.90) ***			
Butterfly rash, n (%)	223 (45.5)			
Discoid rash, n (%)	93 (19.0)			
Photosensitivity, n (%)	190 (38.8)			
Oral ulcers, n (%)	118 (24.1)			
Arthritis, n (%)	241 (49.2)			
Pleurisy, n (%)	45 (9.2)			
Renal disorder, n (%)	184 (37.6)			
Neurological disorder, n (%)	21 (4.3)			
Hematological disorder, n (%)	333 (68.0)			
Immunological disorder, n (%)	359 (73.3)			

* *t*-test; ** χ^2^ test; *** median (interquartile range).

**Table 3:** Genotype and allele frequencies of genotyped SNPs in SLE patients and healthy controls.

SNPs ID	Variable	Genotypes	SLE	Control	*χ^2^*	*p* Value	*OR*	95% *CI*
			n	n				
rs2227314	Genotypes	GG	266	264	0.020	0.990		
		TG	187	187				
		TT	37	38				
	Allele	G	719	715	0.017	0.897	1.013	0.830–1.238
		T	261	263				
	Dominant model	GG	266	264	0.009	0.925	1.012	0.787–1.301
		TT + TG	224	225				
	Recessive model	GG + TG	453	451	0.017	0.897	1.032	0.644–1.652
		TT	37	38				
	Additive model	GG	266	264	0.019	0.890	1.035	0.638–1.678
		TT	37	38				
rs2243115	Genotypes	TT	445	444		1.000 *		
		TG	44	45				
		GG	1	0				
	Allele	T	934	933	0.009	0.922	0.979	0.643–1.492
		G	46	45				
	Dominant model	TT	445	444	0.000	0.992	1.002	0.650–1.546
		GG + TG	45	45				
	Recessive model	TT + TG	489	489	0.000	1.000 **		
		GG	1	0				
	Additive model	TT	445	444	0.000	1.000 **		
		GG	1	0				
rs2243123	Genotypes	TT	422	416		0.250 **		
		TC	62	71				
		CC	6	2				
	Allele	T	906	903	0.010	0.922	1.017	0.728–1.420
		C	74	75				
	Dominant model	TT	422	416	0.219	0.640	1.089	0.762–1.556
		CC + TC	68	73				
	Recessive model	TT + TC	484	487	1.128	0.288 **	0.331	0.067–1.649
		CC	6	2				
	Additive model	TT	422	416	1.065	0.302 **	0.338	0.068–1.685
		CC	6	2				
rs2243131	Genotypes	AA	385	370	1.218	0.544		
		AC	97	109				
		CC	8	10				
	Allele	A	867	849	1.245	0.265	1.166	0.890–1.527
		C	113	129				
	Dominant model	AA	385	370	1.172	0.279	1.179	0.875–1.590
		CC + AC	105	119				
	Recessive model	AA + AC	482	479	0.231	0.631	1.258	0.492–3.214
		CC	8	10				
	Additive model	AA	385	370	0.302	0.583	1.301	0.508–3.332
		CC	8	10				
rs428253	Genotypes	GG	321	321	0.755	0.686		
		GC	156	159				
		CC	13	9				
	Allele	G	798	801	0.073	0.787	0.969	0.771–1.218
		C	182	177				
	Dominant model	GG	321	321	0.002	0.965	0.994	0.764–1.294
		CC + GC	169	168				
	Recessive model	GG + GC	477	480	0.736	0.391	0.688	0.291–1.625
		CC	13	9				
	Additive model	GG	321	321	0.703	0.402	0.692	0.292–1.642
		CC	13	9				
rs4740	Genotypes	GG	172	161	0.545	0.762		
		GA	224	233				
		AA	94	95				
	Allele	G	568	555	0.293	0.588	1.051	0.878–1.257
		A	412	423				
	Dominant model	GG	172	161	0.517	0.472	1.102	0.846–1.436
		AA + GA	318	328				
	Recessive model	GG + GA	396	394	0.009	0.923	1.016	0.740–1.395
		AA	94	95				
	Additive model	GG	172	161	0.177	0.674	1.080	0.755–1.543
		AA	94	95				
rs9807813	Genotypes	CC	321	308	4.740	0.093		
		TC	147	169				
		TT	22	12				
	Allele	C	789	785	0.019	0.892	1.016	0.813–1.269
		T	191	193				
	Dominant model	CC	321	308	0.679	0.410	1.116	0.859–1.450
		TT + TC	169	181				
	Recessive model	CC + TC	468	477	3.026	0.082	0.535	0.262–1.094
		TT	22	12				
	Additive model	CC	321	308	2.415	0.120	0.568	0.277–1.169
		TT	22	12				

* Fisher’s Exact Test; ** Continuity Correction; OR, odds ratio; CI, confidence interval.

**Table 4:** Associations of plasma IL-35 levels with IL-35 genotypes in SLE patients.

SNP ID	Genotypes	*n*	IL-35 Level (pg/mL)			*p* Value
			*M*	*P25*	*P75*	
rs2227314	GG	46	61.51	55.7	72.96	0.538
	TG	25	59.51	52.99	68.57	
	TT	5	64.58	55.4	68.24	
rs2243115	TT	73	61.66	55.07	71.55	0.416
	TG	3	59.51	56.11	59.79	
rs2243123	TT	70	54.23	60.05	71.51	0.315
	TC	5	63.12	68.98	83.05	
	CC	1	61.61	-	-	
rs2243131	AA	65	61.61	55.07	72.08	0.409
	AC	10	57.88	48.19	66.74	
	CC	1	67.51	-	-	
rs428253	GG	45	62.92	55.09	72.08	0.746
	GC	30	59.23	54.14	70.32	
	CC	1	63.72	-	-	
rs4740	GG	29	60.88	54.12	70.70	0.816
	GA	29	58.71	53.7	72.98	
	AA	18	63.52	57.87	72.62	
rs9807813	CC	51	61.61	54.99	71.46	0.666
	TC	21	59.51	54.36	70.78	
	TT	4	69.33	44.32	212.58	

*M*, median; *P25*, percentile 25th, *P75*, percentile 75th, representing the interquartile range.

**Table 5:** Haplotype analysis results among four SNPs in *IL-12A* locus.

Haplotypes	SLE		Control		*χ^2^*	*p* Value	*OR*	95% *CI*
	n	%	n	%				
GGTA	32.34	3.3%	28.92	3.0%	0.188	0.665	1.119	0.672–1.864
GTTA	681.72	69.6%	686.08	70.2%	0.095	0.758	0.969	0.795–1.182
TTCA	63.76	6.5%	62.4	6.4%	0.012	0.912	1.021	0.711–1.465
TTTA	80.07	8.2%	67.12	6.9%	1.200	0.273	1.207	0.861–1.692
TTTC	99.68	10.2%	110.64	11.3%	0.672	0.412	0.887	0.666–1.182

Total *χ^2^* = 1. 927, *df* = 4, *P* = 0.749. All the haplotypes with a frequency <0.03 were ignored in the analysis. OR, odds ratio; CI, confidence interval.

**Table 6:** Haplotype analysis results among three SNPs in *EBI3* locus.

Haplotypes	SLE		Control		*χ^2^*	*p* Value	*OR*	95% *CI*
	n	%	n	%				
CAC	119.38	12.2%	103.00	10.5%	1.433	0.231	1.186	0.897–1.570
CGC	57.14	5.8%	71.86	7.3%	1.744	0.187	0.785	0.548–1.125
GAC	109.22	11.1%	132.13	13.5%	2.388	0.122	0.808	0.616–1.059
GAT	178.17	18.2%	185.74	19.0%	0.161	0.688	0.954	0.760–1.199
GGC	503.26	51.4%	478.01	48.9%	1.511	0.219	1.118	0.936–1.336

Total *χ^2^* = 5.863, *df* = 4, *p* = 0.230. All the haplotypes with a frequency < 0.03 were ignored in the analysis. OR, odds ratio; CI, confidence interval.

## Supplementary Materials

The following are available online at https://www.mdpi.com/2218-273X/9/4/157/s1, Supplementary Tables S1–S7.
