# Supplementary material for: Association between Interleukin 35 Gene Single Nucleotide Polymorphisms and Systemic Lupus Erythematosus in a Chinese Han Population
Source: Biomolecules. 2019 Apr 22;9(4):157. doi: 10.3390/biom9040157 (PMC6523873; doi:10.3390/biom9040157)
Supplement: Supplementary file 1 [file biomolecules-09-00157-s001.pdf]

Table S1 Associations of rs2227314 with clinical manifestations in SLE patients

Manifestations	+/−	Genotypes			χ^2	*P* value	Allele		χ^2	*P* value
		GG	TG	TT			G	T		
Butterfly rash	+	111	94	18	3.387	0.184	316	130	2.65	0.104
	−	155	93	19			403	131		
Discoid rash	+	47	40	6	1.188	0.552	134	52	0.206	0.65
	−	219	147	31			585	209		
Photosensitivity	+	100	80	10	3.571	0.168	280	100	0.032	0.858
	−	166	107	27			439	161		
Oral ulcers	+	63	49	6	1.735	0.42	175	61	0.098	0.754
	−	203	138	31			544	200		
Arthritis	+	133	88	20	0.76	0.684	354	128	0.003	0.957
	−	133	99	17			365	133		
Pleurisy	+	26	17	2	0.747	0.688	69	21	0.552	0.457
	−	240	170	35			650	240		
Renal disorder	+	102	69	13	0.198	0.906	273	95	0.202	0.653
	−	164	118	24			446	166		
Neurological disorder	+	12	8	1		0.949*	32	10	0.179	0.672
	−	254	179	36			687	251		
Hematological disorder	+	183	129	21	2.308	0.315	495	171	0.974	0.324
	−	83	58	16			224	90		
Immunological disorder	+	195	136	28	0.138	0.933	526	192	0.016	0.899
	−	71	51	9			193	69		

+, positive; -, negative; *Fisher's Exact Test; OR, odds ratio; CI, confidence interval

Table S2 Associations of rs2243115 with clinical manifestations in SLE patients

Manifestations	+/−	Genotypes			*P* value	Allele		χ^2	*P* value
		TT	TG	GG		T	G		
Butterfly rash	+	202	20	1	0.804*	424	22	0.104	0.747
	−	243	24	0		510	24		
Discoid rash	+	83	10	0	0.640*	176	10	0.239	0.625
	−	362	34	1		758	36		
Photosensitivity	+	169	20	1	0.221*	358	22	1.665	0.197
	−	276	24	0		576	24		
Oral ulcers	+	105	13	0	0.522*	223	13	0.461	0.497
	−	340	31	1		711	33		
Arthritis	+	218	22	1	0.880*	458	24	0.173	0.678
	−	227	22	0		476	22		
Pleurisy	+	41	4	0	1.000*	86	4	0	1.000**
	−	404	40	1		848	42		
Renal disorder	+	170	14	0	0.701*	354	14	1.042	0.307
	−	275	30	1		580	32		
Neurological disorder	+	21	0	0	0.276*	42	0	1.204	0.273**
	−	424	44	1		892	46		
Hematological disorder	+	301	31	1	0.916*	633	33	0.317	0.574
	−	144	13	0		301	13		
Immunological disorder	+	330	28	1	0.311*	688	30	1.596	0.206
	−	115	16	0		246	16		

+, positive; -, negative; *Fisher's Exact Test; **Continuity Correction; OR, odds ratio; CI, confidence interval

Table S3 Associations of rs2243123 with clinical manifestations in SLE patients

Manifestations	+/−	Genotypes			*P* value	Allele		χ^2	*P* value
		TT	TC	CC		T	C		
Butterfly rash	+	194	26	3	0.809*	414	32	0.166	0.684
	−	228	36	3		492	42		
Discoid rash	+	78	12	3	0.160*	168	18	1.487	0.223
	−	344	50	3		738	56		
Photosensitivity	+	164	23	3	0.804*	351	29	0.006	0.939
	−	258	39	3		555	45		
Oral ulcers	+	100	18	0	0.301*	218	18	0.003	0.959
	−	322	44	6		688	56		
Arthritis	+	206	32	3	0.901*	444	38	0.15	0.698
	−	216	30	3		462	36		
Pleurisy	+	40	4	1	0.443*	84	6	0.111	0.739
	−	382	58	5		822	68		
Renal disorder	+	161	21	2	0.862*	343	25	0.484	0.486
	−	261	41	4		563	49		
Neurological disorder	+	20	1	0	0.620*	41	1	0.996	0.318**
	−	402	61	6		865	73		
Hematological disorder	+	293	37	3	0.161*	623	43	3.567	0.059
	−	129	25	3		283	31		
Immunological disorder	+	310	46	3	0.468*	666	52	0.367	0.545
	−	112	16	3		240	22		

+, positive; -, negative; *Fisher's Exact Test; **Continuity Correction; OR, odds ratio; CI, confidence interval

Table S4 Associations of rs2243131 with clinical manifestations in SLE patients

Manifestations	+/−	Genotypes			*P* value	Allele		χ^2	*P* value
		AA	AC	CC		A	C		
Butterfly rash	+	172	48	3	0.687*	392	54	0.267	0.605
	−	213	49	5		475	59		
Discoid rash	+	79	13	1	0.244*	171	15	2.704	0.1
	−	306	84	7		696	98		
Photosensitivity	+	152	35	3	0.879*	339	41	0.334	0.563
	−	233	62	5		528	72		
Oral ulcers	+	88	28	2	0.432*	204	32	1.254	0.263
	−	297	69	6		663	81		
Arthritis	+	187	49	5	0.692*	423	59	0.469	0.494
	−	198	48	3		444	54		
Pleurisy	+	37	8	0	0.928*	82	8	0.678	0.41
	−	348	89	8		785	105		
Renal disorder	+	145	36	3	1.000*	326	42	0.008	0.929
	−	240	61	5		541	71		
Neurological disorder	+	18	2	1	0.212*	38	4	0.029	0.866**
	−	367	95	7		829	109		
Hematological disorder	+	263	66	4	0.551*	592	74	0.359	0.549
	−	122	31	4		275	39		
Immunological disorder	+	280	73	6	0.905*	633	85	0.249	0.617
	−	105	24	2		234	28		

+, positive; -, negative; *Fisher's Exact Test; **Continuity Correction; OR, odds ratio; CI, confidence interval

Table S5 Associations of rs428253 with clinical manifestations in SLE patients

Manifestations	+/−	Genotypes			χ^2	*P* value	Allele		χ^2	*P* value
		GG	GC	CC			G	C		
Butterfly rash	+	152	66	5	1.345	0.51	370	76	1.269	0.26
	−	169	90	8			428	106		
Discoid rash	+	65	25	3		0.452*	155	31	0.551	0.458
	−	256	131	10			643	151		
Photosensitivity	+	130	55	5	1.216	0.545	315	65	0.882	0.348
	−	191	101	8			483	117		
Oral ulcers	+	81	34	3		0.685*	196	40	0.541	0.462
	−	240	122	10			602	142		
Arthritis	+	160	73	8	1.206	0.547	393	89	0.007	0.933
	−	161	83	5			405	93		
Pleurisy	+	33	11	1		0.508*	77	13	1.116	0.291
	−	288	145	12			721	169		
Renal disorder	+	122	61	1		0.074*	305	63	0.821	0.365
	−	199	95	12			493	119		
Neurological disorder	+	14	6	1		0.623*	34	8	0.007	0.935
	−	307	150	12			764	174		
Hematological disorder	+	215	110	8		0.630*	540	126	0.166	0.684
	−	106	46	5			258	56		
Immunological disorder	+	231	119	9		0.538*	581	137	0.461	0.497
	−	90	37	4			217	45		

+, positive; -, negative; *Fisher's Exact Test; OR, odds ratio; CI, confidence interval

Table S6 Associations of rs4740 with clinical manifestations in SLE patients

Manifestations	+/−	Genotypes			χ^2	*P* value	Allele		χ^2	*P* value
		GG	GA	AA			G	A		
Butterfly rash	+	76	105	42	0.316	0.854	257	189	0.038	0.846
	−	96	119	52			311	223		
Discoid rash	+	25	50	18	3.838	0.147	100	86	1.659	0.198
	−	147	174	76			468	326		
Photosensitivity	+	73	87	30	2.838	0.242	233	147	2.87	0.09
	−	99	137	64			335	265		
Oral ulcers	+	39	55	24	0.322	0.851	133	103	0.328	0.567
	−	133	169	70			435	309		
Arthritis	+	86	107	48	0.358	0.836	279	203	0.002	0.962
	−	86	117	46			289	209		
Pleurisy	+	22	15	8	4.396	0.111	59	31	2.347	0.126
	−	150	209	86			509	381		
Renal disorder	+	75	89	20	13.759	0.001	239	129	11.804	0.001
	−	97	135	74			329	283		
Neurological disorder	+	6	12	3		0.637*	24	18	0.012	0.913
	−	166	212	91			544	394		
Hematological disorder	+	106	165	62	6.683	0.036	377	289	1.561	0.212
	−	66	59	32			191	123		
Immunological disorder	+	121	168	70	1.16	0.56	410	308	0.808	0.369
	−	51	56	24			158	104		

+, positive; -, negative; *Fisher's Exact Test; OR, odds ratio; CI, confidence interval

Table S7 Associations of rs9807813 with clinical manifestations in SLE patients

Manifestations	+/−	Genotypes			χ^2	*P* value	Allele		χ^2	*P* value
		CC	TC	TT			C	T		
Butterfly rash	+	137	76	10	3.309	0.191	350	96	2.16	0.142
	−	184	71	12			439	95		
Discoid rash	+	56	35	2		0.142*	147	39	0.32	0.572
	−	265	112	20			642	152		
Photosensitivity	+	128	56	6	1.418	0.492	312	68	1.006	0.316
	−	193	91	16			477	123		
Oral ulcers	+	75	38	5	0.364	0.834	188	48	0.143	0.705
	−	246	109	17			601	143		
Arthritis	+	153	75	13	1.359	0.507	381	101	1.297	0.255
	−	168	72	9			408	90		
Pleurisy	+	35	8	2		0.134*	78	12	2.394	0.122
	−	286	139	20			711	179		
Renal disorder	+	126	54	4	3.958	0.138	306	62	2.621	0.105
	−	195	93	18			483	129		
Neurological disorder	+	15	6	0		0.928*	36	6	0.757	0.384
	−	306	141	22			753	185		
Hematological disorder	+	213	106	14	1.731	0.421	532	134	0.526	0.468
	−	108	41	8			257	57		
Immunological disorder	+	232	113	14	2.178	0.337	577	141	0.038	0.846
	−	89	34	8			212	50		

+, positive; -, negative; *Fisher's Exact Test; OR, odds ratio; CI, confidence interval
